# Overcoming limitations of current antiplatelet drugs: A concerted effort for more profitable strategies of intervention

**DOI:** 10.3109/07853890.2011.582137

**Published:** 2011-08-05

**Authors:** Matteo Nicola Dario Di Minno, Anna Guida, Marina Camera, Susanna Colli, Giovanni Di Minno, Elena Tremoli

**Affiliations:** 1 Department of Clinical and Experimental Medicine, Regional Reference Centre for Coagulation Disorders, ‘Federico II’ University, Naples, Italy; 2 Department of Pharmacological Sciences, Università degli Studi di Milano, Milan, Italy; 3 Centro Cardiologico Monzino IRCCS, Milan, Italy

**Keywords:** Aspirin failure, cardiovascular prevention, clopidogrel failure, new antiplatelet drugs

## Abstract

Platelets play a central role in the pathophysiology of atherothrombosis, an inappropriate platelet activation leading to acute ischemic complications (acute myocardial infarction, ischemic stroke). In view of this, platelets are a major target for pharmacotherapy. Presently, the main classes of antiplatelet agents approved for the use in such complications are aspirin and fhienopyridines. Although antiplatelet treatment with these two types of drugs, alone or in combination, leads to a significant reduction of non-fatal myocardial infarction (−32%), non-fatal stroke (−25%), and of cardiovascular death (−17%), a residual risk persists.

Newer antiplatelet agents have addressed some, but not all, these limitations. Vis-à-vis their net clinical benefit, the higher potency of some of them is associated with a rise in bleeding complications. Moreover, newer fhienopyridines do not show advantages over and above the older ones as to reduction of stroke. A concerted effort that takes into consideration clinical, genetic, and laboratory information is increasingly recognized as a major direction to be pursued in the area. The well-established road signs of clinical epidemiology will provide major information to define newer potentially useful targets for platelet pharmacology.

## Introduction

Thrombotic (ischemic) complications of atherosclerosis (acute coronary syndrome (ACS), acute myocardial infarction (AMI), ischemic stroke) are the leading causes of morbidity and mortality in Western countries. Following atherosclerotic plaque disruption/endothelial cell detachment, circulating platelets, exposed to a highly thrombogenic environment, become activated (1). As shown in Table I, a series of soluble agonists (ADP, thromboxane A_2_ (T×A_2_), serotonin (5-HT), and thrombin) recruit and activate additional platelets (2,3). Upon activation, glycoprotein (Gp) IIb/IIIa (α_IIb_β_3_ integrin) mediates platelet aggregation and spreading by means of fibrinogen bridges, which, once converted to fibrin, ultimately contribute to thrombus stabilization (4). This leads to the formation of platelet-rich thrombi that, occluding the arterial lumen and impairing blood-flow and oxygen supply, cause acute ischemia (5).

**Table I:** Platelet activation: agonists and signal transduction.

Agonist	Receptor(s)	Effect(s)	Comments
ADP	Gaq-coupled P_2_Y_1_; Gai-coupled P_2_Y_12_	P_2_Y_1_: shape change; P_2_Y_12_: aggregation	P_2_Y_12_ amplifies aggregation induced by 5-HT, T×A_2_, and thrombin
T×A_2_	TPa;TPb (secondary)	Shape change; platelet recruitment; platelet aggregation	In endothelial cells, COX-1-derived PGH_2_ is converted into PGI_2_, a strong antiaggregating and vasodilating agent
Thrombin	PAR-1; PAR-4 (secondary); GpIbα	Shape change; ADP and T×A_2_ secretion; P-selectin expression; α_IIb_β_3_ integrin receptor; platelet activation	PAR-1 and P_2_Y_12_ cross-reaction (via Gαq and Gai-coupled receptors)
Serotonin	5HT-2A receptors	Shape change; platelet recruitment; retention of fibrinogen and thrombospondin on the platelet surface	Implicated in shear-induced platelet aggregation and thrombus propagation

The transduction of the ADP signal involves its interaction with two platelet receptors belonging to the P_2_ purinergic family, the Gαq-coupled receptor P_2_Y_1_ and the Gαi-coupled receptor P_2_Y_12_. The concomitant activation of both the Gαq and Gαi pathways by ADP is needed for platelet aggregation to occur. Signaling from the P_2_Y_1_ receptor causes platelet shape change and rapid transient aggregation, whereas the signaling from the P_2_Y_12_ receptor facilitates sustained irreversible aggregation and stimulates surface expression of the pro-inflammatory P-selectin (6). In addition, the P_2_Y_12_ receptor plays a critical role in the amplification of platelet aggregation induced by agents other than ADP, including 5-HT,T×A_2_, and thrombin. Together, these contribute to thrombus growth and stability (5).

**Key messages**

- Platelets are a major target for pharmacotherapy in cardiovascular disease.
- In spite of a significant antithrombotic effect, current antiplatelet drugs have major limitations.
- Newer antiplatelet agents have addressed some but not all the limitations of the current antiplatelet drugs.

T×A_2_, generated from arachidonic acid by cyclooxygenase 1 (COX-1) and T× synthase, further amplifies platelet activation. COX-1 converts arachidonic acid into prostaglandin endoperoxides PGG_2_ and PGH_2_, the latter being, in turn, transformed by T× synthase into T×A_2_, a potent amplifier of platelet aggregation with vasoconstrictive properties. At the site of the atheroma rupture, platelet-released T×A_2_ leads to downstream micro-vessel contraction and thrombus propagation (7).

In addition to its role in coagulation, thrombin at extremely low concentrations is one of the major platelet activators (8). Human platelets express two cell surface G-protein-coupled protease-activated receptors (PARs) for thrombin: PAR-1 and PAR-4. By binding the hirudin-like extracellular amino terminal domain (the so-called thrombin receptor), thrombin activates platelets and smooth muscle cells, thus promoting platelet pro-coagulant activity, shape change, secretion and release of agonists (ADP and T×A_2_), expression of P-selectin, activation of the α_IIb_β_3_ integrin receptor, and aggregation. Thrombin also binds to GpIbα on the surface of platelets, thought to act as a co-factor that localizes the enzyme on the platelet surface and accelerates the hydrolysis of PAR-1 (9). PAR-1 and P_2_Y_12_ cross-react in platelet activation, and Gαq and Gαi-coupled receptors are involved in this process. Thrombin-dependent platelet aggregation is mediated in part by secreted ADP, acting on the Gαi-linked ADP receptor. By blocking Gαq via PAR-1 and Gβi via P_2_Y_12_, combined inhibition of thrombin and P_2_Y_12_ receptors leads to a synergistic inhibitory effect on thrombin-induced platelet aggregation (10).

5-HT is a vasoconstrictor agent that binds to 5HT-2A receptors and amplifies the platelet response by stimulating shape change and enhancing platelet recruitment at sites of injury (3). It may also play a pro-coagulant role by promoting the retention of fibrinogen and thrombospondin on the platelet surface. Intraplatelet 5-HT stores are implicated in shear-induced platelet aggregation and thrombus propagation (3).

## Antiplatelet agents in clinical practice: efficacy, safety, limitations (Figure 1)

Three main classes of antiplatelet agents are licensed for treatment of atherothrombosis: acetyl salicylic acid (aspirin), P_2_Y_12_ inhibitors (ticlopidine, clopidogrel, prasugrel, cangrelor, ticagrelor), and GpIIb/ Ilia receptor antagonists (abciximab, eptifibatide, and tirofiban) (11). While the last-mentioned are only employed intravenously in ACS in combination with other antithrombotic agents (their effect being rather unpredictable when administered orally), the former two classes are widely used chronically.

**Figure 1:**
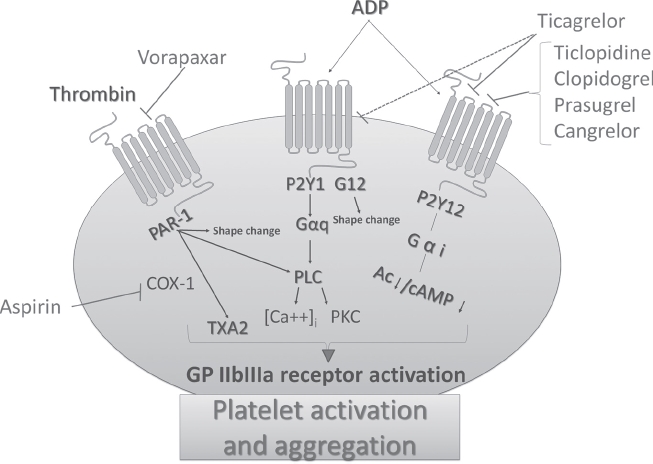
Platelet activation pathways and drug molecular targets. Thrombin binds to PAR-1 receptor, which leads to shape change, phospholipase C (PLC) activation, thromboxane A2 (T×A2) generation, and activation of the glycoprotein (GP) IIb/IIIa receptor, resulting in sustained platelet aggregation. Cyclooxygenase (COX)-l catalyzes the production of T×A2, a potent platelet aggregator, generated by platelets activated by thrombin and other agonists. Adenosine 5'-diphosphate (ADP) binds to its 7-transmembrane domain receptors, P2Y1 and P2Y12, to activate platelets. P2Y1 is coupled to Gαq and G12. Gαq is linked to a signaling pathway involving PLC activation, resulting in a rise in the intracellular calcium concentration ([Ca+2]i) and protein kinase C (PKC) activation, leading to GP IIb/IIIa activation and transient platelet aggregation. G12 mediates platelet shape change. P2Y12 is linked to Gai-coupled signaling cascades associated with adenylcyclase (Ac) down-regulation and decreased cyclic-3',5'-monophosphate (cAMP) production, which mediates GP IIb/IIIa receptor activation, leading to sustained platelet aggregation.

### Aspirin

Owing to its efficacy and favorable cost-effectiveness, aspirin is the mainstay treatment for all atherothrombotic conditions. The efficacy of aspirin lies in its ability to inhibit irreversibly platelet COX-1 (by acetylating a serine located near the active site of the enzyme) and, in turn, T×A_2_ formation (12). A recent meta-analysis by the Antithrombotic Trialists’ Collaboration (13) assessed the role of aspirin in primary (95,000 subjects at low cardiovascular risk) and secondary prevention (17,000 patients at medium/high risk) and showed that, while in high-risk conditions the advantages of aspirin outweigh the inherent bleeding hazard, in primary prevention aspirin is associated with an absolute benefit of 0.06%/year, too exiguous as compared to the 0.03% increase in major bleedings.

### Ticlopidine

Ticlopidine was the first agent of the thienopyridine class shown both to prevent the interaction of ADP with its platelet purinergic receptor and to determine the inhibition of the binding of fibrinogen to the α_IIb_β_3_ integrin (14). In subjects with a history of cerebrovascular events, ticlopidine was superior to placebo and to aspirin in the reduction of stroke, AMI, or vascular death (15). In addition, the combination of ticlopidine with aspirin was successful in ACS patients undergoing percutaneous coronary intervention (PCI) with stent implantation (16). Diarrhea, neutropenia, aplastic anemia, and thrombotic thrombocytopenic purpura are the main limitations for a wide-spread use of ticlopidine.

### Clopidogrel

The thienopyridine prodrug clopidogrel irreversibly binds the P_2_Y_12_ platelet receptor after a two-step activation by cytochrome P450 (CYP) liver isoenzymes. While the CAPRIE trial (about 20,000 subjects) (17) and a Cochrane systematic review (18) showed that in patients with a history of AMI, stroke, or symptomatic peripheral arterial disease (PAD), clopidogrel administered alone was only marginally superior to aspirin (RRR 8.7; *P*= 0.043) in preventing vascular events, a significantly higher efficacy has been shown for the ‘dual antiplatelet therapy'. The CURE (19) and the CREDO (20) studies established the superiority of clopidogrel in combination with aspirin versus aspirin alone in ACS and in ACS with PCI, respectively.

## Low response to aspirin or clopidogrel

Although antiplatelet treatment in high-risk patients significantly lowers non-fatal AMI (32%), non-fatal stroke (25%), and cardiovascular death (17%), a residual vascular risk persists. After an AMI or a stroke, in a long-term follow-up, 10%-20% of patients with a history of an ischemic event develop recurrent events in spite of their antiplatelet treatment (21). Compared to aspirin-sensitive patients, the residual platelet reactivity in subjects on antiplatelet therapy correlates with a higher cardiovascular risk and predicts recurrent thrombotic events (22). In the HOPE study, aspirin-treated patients in the highest quartile of the urinary excretion of T×A_2_ metabolite had a 1.8-fold higher vascular risk than those in the lowest quartiles (23). A meta-analysis (24) evaluated the relation between a low laboratory response to clopidogrel and the clinical outcome in ≈4,500 patients with coronary artery disease (CAD) and PCI. Several methods were used to assess platelet response to therapy, and 26.4% of patients showed as low responders to clopidogrel. These patients showed a higher risk of death/ischemic events as compared to that of patients in whom a normal response was documented.

Although often secondary to a poor compliance (25), a low treatment response ('failure') may be due, at least in part, to an incomplete inhibition of platelet function (26). Concomitant clinical conditions (diabetes mellitus, inflammation, hypercoagulable states, low fibrinolytic potential), high pretreatment platelet reactivity (observed in ACS or in subjects with high BMI), an increased platelet turn-over, and the simultaneous administration of some drugs (drug interaction and/or inadequate drug absorption) are common, major determinants of a low response to clopidogrel or to aspirin (21,27). The possibility that, in addition to such conditions, genetic variations play a role in a low response to aspirin or to clopidogrel has also been documented. Different haplotypes of COX-1 significantly correlate with the response to aspirin and with T×A_2_ generation in patients with stable coronary artery disease (28).

A variety of polymorphisms in the CYP2C19 gene (most often the CYP2C19*2), associated with a 20%-25% production of inactive metabolite, diminish the response to clopidogrel. Among subjects under treatment with clopidogrel for a previous vascular event, carriers of these polymorphisms have a 50% higher risk of cardiovascular death, AMI, or stroke (29). Among clopidogrel-treated patients, carriers of at least one allele associated with the loss of or a reduced function in the CYP2C19 gene had a higher than normal occurrence of fatal and non-fatal coronary thrombotic events, as well as of stent thrombosis (29). In the same study population, polymorphic alleles of a gene modulating clopidogrel absorption (ABCB1) have been associated with a higher rate of cardiovascular events at 1-year follow-up as compared to wild-type subjects.

## Overcoming limitations of aspirin

### Changing doses and schedules of aspirin

Because inhibition of platelet aggregation by aspirin is irreversible, the possibility of monitoring the entry of newly formed platelets into the circulation (i.e. the platelet turn-over) was documented by measuring the recovery of TxB_2_ biosynthesis after aspirin ingestion (30). The data show that platelets with intact cyclo-oxygenase activity can be detected into the circulation as early as 4-6 h after aspirin ingestion. A higher than normal rate of entry of platelets into the circulation has been documented in diabetic angiopathy or in patients with coronary artery bypass (31). Indeed, schedules of aspirin which may suffice in normal subjects are not effective in patients with diabetic angiopathy, presumably because these patients have a high rate of entry of new platelets into the circulation (27). A long-lasting suppression of thromboxane biosynthesis may be achieved by repeated low-dose administrations or by slow-release preparations of aspirin. The relevance of an accelerated platelet turn-over as to ‘aspirin resistance’ has been confirmed in a series of clinical settings (32,33).

Attempts to improve the efficacy of aspirin by using larger doses have been associated with contrasting results (34). Moreover, a significant higher bleeding risk (mainly gastrointestinal) may occur with higher aspirin doses. Thus, increasing the dose of aspirin would expose the patient to a doubtful clinical advantage (35).

## Overcoming limitations of clopidogrel

### Changing doses of clopidogrel

In spite of the greater antithrombotic efficacy of higher doses of clopidogrel, thrombotic events still occur in 4.2% of patients (36).

Whereas a vasodilator-stimulated phosphoprotein (VASP)-tailored clopidogrel loading dose has been found to improve the clinical outcome (37) and to reduce the rate of early stent thrombosis after PCI (38), the GRAVITAS trial has been designed to evaluate only the efficacy and safety of VASP-tailored clopidogrel-maintaining regimens (39). Early results of the study indicate that, compared with standard-dose, high-dose clopidogrel only achieved a modest pharmacodynamic effect in PCI patients with high residual platelet activity. Moreover, 6-month higher-dose clopidogrel did not reduce the rate of cardiovascular death, non-fatal MI, or stent thrombosis, nor did it increase severe or moderate bleeding. Thus the GRAVITAS study does not support a treatment strategy of high clopidogrel dose in PCI patients with high residual activity, identified by a single platelet function test.

Further strategies have been evaluated to overcome clopidogrel limitations. Ω-3 Ethyl esters (1 g/day for 1 month) added to standard dual antiplatelet therapy significantly lowered maximal platelet aggregation in response to ADP and VASP phosphorylation as well (40).

Cilostazol, selectively targeting phosphodiesterase type 3 (PDE3) and, then, determining intracellular cAMP accumulation, inhibits platelet aggregation (41). In diabetic patients on standard dual antiplatelet therapy, adjunctive treatment with cilostazol enhances inhibition of platelet P_2_Y_12_ signaling (42). A Cochrane review (43), in which two randomized studies on stroke prevention were summarized, documented that, compared with aspirin, cilostazol was associated with a significantly lower risk of vascular events (6.77% versus 9.39%; RR 0.72; 95% CI 0.57-0.91, composite outcome), and with a lower risk of hemorrhagic stroke (0.53% versus 2.01%; RR 0.26; 95% CI 0.13-0.55). In terms of outcome of safety, cilostazol was associated with significantly fewer adverse events (8.22% versus 4.95%; RR 1.66; 95% CI 1.51-1.83) than aspirin. In the SILOAM phase IV study (Clini-calTrials.gov Identifier: NCT01261832), a triple antiplatelet therapy (cilostazol plus aspirin and clopidogrel) is compared (at 1 month and at 6 months) with the standard dual antiplatelet treatment (ASA and clopidogrel) in 951 ACS subjects (expected number) undergoing PCI and drug eluting-stent implantation. The primary efficacy end-point is the occurrence of major cardiovascular and cerebrovascular events (total death, non-fatal myocardial infarction, repeat revascularization, stroke). The end of the study is expected by January 2013.

## Exploring newer antiplatelet strategies (Table II)

### Prasugrel

Prasugrel, a prodrug of the thienopyridine family, after a rapid one-step conversion into a highly bioavailable metabolite, causes an irreversible block of the P2Y12 ADP receptor. Because of a distinct chemical structure, the conversion to its active metabolite is less dependent on specific cytochrome P450 enzymes than that of other thienopyridines (44). A phase I study (45) evaluated the inhibition of ADP-induced platelet aggregation by prasugrel (60 mg loading and 10 mg repeated doses) as compared to clopidogrel (300 mg loading dose and 75 mg repeated doses). In platelets exposed to ADP, inhibition of the aggregation and the onset of the antiplatelet effect were higher for prasugrel than for clopidogrel (maximal inhibition achieved 30-60 min and 4-6 hours after prasugrel and clopidogrel, respectively). Of interest, the number of ‘non-responders’ was significantly lower with a 60-mg loading dose of prasugrel than with a 300-mg loading dose of clopidogrel (0% versus 42%).

**Table II:** Molecular targets of newer and emerging antiplatelet therapies.

Drug	Mechanism	Comment
Prasugrel	P_2_Y_12_ receptor inhibition	Irreversible inhibition; orally active
Cangrelor	Adenosine triphosphate analog with a high affinity for the P_2_Y_12_ receptor	Reversible inhibition; intravenous
Ticagrelor	P_2_Y_12_ and (partly) P_2_Y_1_ receptor inhibition	Reversible inhibition; orally active
Elinogrel	P_2_Y_12_ receptor inhibition	Reversible; oral and intravenous
Vorapaxar (E530348)	PAR-1 inhibitor	No effect on thrombin-induced fibrin production, orally active
Atopaxar (E5555)	Low-molecular-weight PAR-1 inhibitor	Inhibition of expression of the inflammatory markers (sCD40L and interleukin 6 and the expression of P-selectin), orally active
Sarpogrelate	Selective inhibitor of serotonin (5HT-2A) platelet receptors	Orally active
DZ-697b	Inhibits collagen and ristocetin-mediated platelet adhesion and aggregation	Orally active
Gas6 (growth arrest-specific gene 6)	Vitamin K-dependent protein; a polyclonal anti-Gas6 antibody lowers platelet thromboembolism induced by the intravenous injection of collagen plus epinephrine and aggregation by ADP	Stored in platelet a granules, released upon activation. Through its carboxy-terminal domains it interacts with the membrane receptor tyrosine kinases (RTKs) of the TAM family (Tyro3, Axl, MerTK); through its vitamin K-dependent Gla module it interacts with phosphatidylserine-containing membranes
Matrix metalloproteinases (MMPs)	By potentiating PI3K activation, MMP-2 amplifies platelet aggregation regardless of the presence of aspirin and of P_2_Y_12_ receptor antagonists	Involved in tissue remodeling and in the progression of atherosclerosis, MMP-2 is present in platelet cytosol and released upon aggregation
CD40 ligand (CD40L; CD 154)	On activated platelets an exodomain (soluble CD40 ligand, sCD40L) is released and binds to α_IIb_β_3_ integrin, thus promoting thrombus stabilization and blunting platelet reactivity	Transmembrane protein expressed on the surface of activated platelets. Shedding of sCD40L from the surface of activated platelets can be prevented by an anti-CD40L antibody (G28-5)

A phase II dose-ranging trial, the JUMBO-TIMI 26 (46), compared clopidogrel and prasugrel regimens in 900 patients undergoing elective or urgent PCI plus stenting. Patients were randomized to one of three combinations of prasugrel loading and maintenance doses: 40 mg and 7.5 mg/d, 60 mg and 10 mg/d, and 60 mg and 15 mg/d, or to the standard clopidogrel regimen (300 mg and 75 mg/d). While the thrombolysis in myocardial infarction (TIMI) major bleeding was similar between prasugrel and clopidogrel groups, subjects receiving prasugrel showed a lower incidence of the composite end-point of 30-day major adverse cardiac events. Likewise, significantly lower rates of coronary target vessel thrombosis were seen in prasugrel-treated patients. The PRINCIPLE-TIMI 44 (47) was a randomized, double-blind, two-phase cross-over trial of prasugrel compared with high-dose clopidogrel in 201 patients undergoing a planned PCI. In the first phase, a 60-mg prasugrel loading dose was compared with a 600-mg loading dose of clopidogrel. After the loading dose, the subjects received prasugrel 10 mg or clopidogrel 150 mg for 14 days, and then they were crossed over to the alternative treatment for an additional 14 days. The primary end-point was the inhibition of platelet aggregation at 6 hours following 20 μmol/L ADP. After the loading dose, the inhibition of platelet aggregation at 6 hours was significantly greater in the patients receiving prasugrel than in those on clopidogrel. In addition, patients on prasugrel showed more consistent levels of platelet inhibition, a lower interindividual variability, and a reduced incidence of low response as compared to those on clopidogrel. The same figures were confirmed after 14 days of maintenance therapy. Thus, both the 60 mg loading dose and the maintenance dose of 10 mg of prasugrel were superior to clopidogrel regimen in inhibiting P_2_Y_12_-dependent platelet aggregation. As to safety findings, no TIMI major bleeding event occurred after the loading dose in either group, whereas two subjects (2.0%) in the prasugrel group and no subject in the clopidogrel group experienced a TIMI minor bleeding episode before the cross-over.

The TRITON-TIMI 38 (48) was a randomized, double-blind, parallel-group, multinational phase III trial comparing the efficacy and safety of prasugrel and clopidogrel in 13,608 subjects with ACS undergoing PCI and coronary stenting. Patients were randomized to receive prasugrel (60-mg loading dose followed by a 10-mg/day maintenance dose) or clopidogrel (300-mg loading dose followed by a 75-mg/day maintenance dose) for a mean of 14.5 months. The primary efficacy end-point (death from CV causes, non-fatal MI, non-fatal stroke) occurred in 643 patients (9.9%) receiving prasugrel and in 781 patients (12.1%) receiving clopidogrel (hazard ratio (HR) 0.81; 95% CI 0.73-0.90; *P*= 0.001). An early significant difference in the primary end-point was also documented at the 3-day pre-specified time point, a finding consistent with a more rapid onset of the antiplatelet activity of prasugrel (43). Such difference was maintained from the third day to the end of the study. The difference in the primary end-point was found both among patients with unstable angina and non-STEMI and STEMI subjects, the reduction in AMI being the major determinant of such difference. In addition, regardless of the type of stent, a significant 52% reduction of stent thrombosis was found in the prasugrel group (49).

**Table III:** Efficacy and safety of newer antiplatelet drugs. Results from phase III studies.

Study (ref)	
TRITON-TIMI 38 (48), 13,608 subjects:	
Study design	Prasugrel (60-mg loading dose (LD) 10-mg maintenance dose (MD) versus Clopidogrel (300 mg LD 75 mg MD)
Efficacy end-point	9.9% versus 12.1%; *P* = 0.001
Safety end-point	2.4% versus 1.8%; *P* = 0.03
CHAMPION-PCI (52), 8,887 subjects:	
Study design	Cangrelor (bolus 30 μg/kg + infusion 4 μg/kg/min) + Clopidogrel (600 mg LD) versus Placebo + Clopidogrel (600 mg LD)
Efficacy end-point	7.5% versus 7.9%; *P*= 0.59
Safety end-point	0.4% versus 0.3%; *P*= 0.39
CHAMPION-PLATFORM (53), 2,654 subjects:	
Study design	Cangrelor (bolus 30 μg/kg or 4 μg/kg/min infusion for a 2 h) + Clopidogrel (600 mg LD) versus Placebo + Clopidogrel (600 mg LD)
Efficacy end-point	7.0% versus 8.0%; *P*= 0.17
Safety end-point	3.5% versus 5.5%; *P<* 0.001
The PLATO trial (53), 18,624 subjects:	
Study design	Ticagrelor (180 mg LD + 90 mg twice a day MD) or Clopidogrel (300-600 mg LD + 75 mg MD)
Efficacy end-point	9.8% versus 11.7%; *P*= 0.001
Safety end-point	11.6% versus 11.2%; *P*= 0.46

Efficacy end-point: CV death/non-fatal Ml/non-fatal stroke. Safety end-point: major bleeding.

Vis-à-vis a 2.2% reduction in fatal and non-fatal ischemic events, both TIMI major and minor bleeding episodes were 1.2% more frequent with prasugrel than with clopidogrel. The TIMI major bleeding was observed in 2.4% of subjects in the prasugrel group and in 1.8% in the clopidogrel group. The rate of life-threatening bleeding (1.4% versus 0.9%; P=0.01) and of fatal bleeding (0.4% versus 0.1%; *P* = 0.002) was greater in the prasugrel group than in the clopidogrel group, with maximal bleeding risk in patients with a history of stroke/TIA (in whom this drug should be avoided, being associated with increased intracranial hemorrhage), in elderly patients (aged ≥ 75 years) and in those with a body-weight < 60 kg. On the other hand, based on TIMI major bleedings (key safety end-point of the trial), prasugrel should also be used with caution in candidates to Coronary Artery Bypass Graft (CABG), > 50% of total bleedings being reported in this setting. Together, in a risk/benefit analysis, prasugrel was 13% better than clopidogrel (HR 0.87; 95% CI 0.79-0.95; *P* = 0.004), maximal clinical benefit being found in patients with diabetes, with coronary stents, or with recurrent events (RRR 30%). Whether prasugrel is safer and better than clopidogrel in reducing the risk of cardiovascular death, MI, or stroke, in patients with ACS who are medically managed, and in whom no revascularization is planned, will be evaluated in the TRILOGY ACS study, a phase III multicenter, double-blind, randomized, controlled trial including approximately 10,000 patients (50).

### Cangrelor

Cangrelor, an adenosine triphosphate (ATP) analog with a high affinity for the P_2_Y_12_ receptor (35), does not need conversion, being immediately active following infusion (half-life of 3-6 min). In the STEP-AMI trial, 92 ACS patients (51) treated with aspirin and heparin were randomized to receive cangrelor (280 μg/kg/min) alone, full-dose tissue plasminogen activator (t-PA) alone, or cangrelor (35, 140, or 280 μg/kg/min) in combination with half-dose t-PA. A 60-min coronary patency similar to that of full-dose t-PA alone and a greater patency than with cangrelor alone was found in patients receiving the combination of cangrelor and half-dose t-PA. Although in two randomized controlled clinical phase III trials on ACS patients requiring PCI (CHAMPION PCI, CHAMPION PLATFORM) (52,53), cangrelor did not show superiority over clopidogrel, this drug is currently investigated as a bridge to CABG surgery (ClinicalTrials.gov Identifier: NCT00767507).

### Ticagrelor

Ticagrelor, an orally active cyclopentyl-triazolo-pyrimidine, binds to domains of the P_2_Y_12_ receptor other than those recognized by ADP (the 1, 2, and 7 transmembrane domains, the extracellular loop 2, and the N-terminal domain), determining a potent and rapid non-persistent receptor conformational change. After the occupancy of P_2_Y_12_, ADP-catalyzed conversion of cAMP from ATP, dephosphorylation of phosphorylated VASP, and activation of phosphoinositide 3-kinase are blocked. The net result is a reduced exposure of fibrinogen-binding sites on the α_IIb_β_3_ integrin receptor and, in turn, the inhibition of platelet aggregation. Inhibition of ADP-mediated constriction of vascular smooth muscle and enhancement of adenosine-induced coronary blood-flow are also reported. After oral administration, ticagrelor is rapidly absorbed and does not require hepatic biotransformation to be pharmacologically active. However, ticagrelor is also metabolized to an equipotent, active metabolite (AR-C124910XX) by CYP_3_A_4_ enzymes. Being both ticagrelor and AR-C124910XX-excreted by the intestinal route, no dose adjustment is needed in kidney failure. On the other hand, the concomitant use of CYP_3_A_4_ inhibitors/inducers as well as a significant liver dysfunction may be of concern for its use (54). After pharmacodynamic evaluations (55,56), a 90-mg twice daily dose of ticagrelor has been chosen to optimize its efficacy, safety, and tolerability. A loading dose of 180-270 mg may minimize intersubject variability as to initial inhibition in platelet aggregation and may be appropriate in ticagrelor-naive patients with ACS or in preparation for PCI.

In 174 subjects with a recent coronary artery disease receiving 75-100 mg/day aspirin (92 also under ticagrelor 180-mg load and 90 mg/twice daily maintenance dose, and 82 also under clopidogrel 600-mg load and 75 mg/d maintenance dose) the genotyping of the cytochrome P450 (CYP) 2C19 (*1,*2,*3,*4,*5,*6,*7,*8,*17) was performed. In addition, platelet function was measured by aggregometry, VerifyNow P_2_Y_12_ assay, and VASP assay at pre-dose, 8 hours post-loading, and during maintenance. There was no significant effect of the genotype on platelet function during aspirin therapy alone. On the other hand, irrespective of the 2C19 genotype, of the metabolizer status, and of the assays employed, subjects on ticagrelor showed a lower platelet reactivity than did those on clopidogrel (P<0.01). This is consistent with a genotype-independent better pharmacodynamic effect of ticagrelor as compared to clopidogrel (57).

The DISPERSE trial (200 subjects with stable atherosclerotic disease) (54) showed ticagrelor (100 mg/b.i.d., 200 mg/b.i.d., or 400 mg/d) to inhibit platelet aggregation more rapidly and effectively and with less variability than clopidogrel (75 mg/d). The DISPERSE-2 trial (58) (990 NSTEMI patients) showed that ticagrelor, added to standard medical treatment, has a safety profile similar to clopidogrel with a better profile as to the incidence of AMI, silent AMI, severe recurrent ischemia, stroke, and death. However, the study was not powered to detect differences in efficacy end-points (size), nor was the duration of the drug exposure appropriate (52).

The PLATO trial was a phase III, multicenter, randomized, double-blind, double-dummy study that evaluated the efficacy and safety of ticagrelor and of clopidogrel in lowering the risk of vascular events in more than 18,000 NSTEMI or STEMI patients (59). Within 24 hours after a diagnosis of ACS, the patients were randomized to receive ticagrelor 90 mg/twice daily or clopidogrel 75 mg once daily for 6-12 months. Before starting the maintenance dose, each subject received a loading dose of 180 mg ticagrelor or 300 mg clopidogrel, depending on the group of randomization. All subjects received concomitant treatment with aspirin 75-100 mg/day. The primary outcome of the study was the time to the first occurrence of cardiovascular or cerebrovascular death, non-fatal MI, or non-fatal stroke. The primary safety outcome was the time to the first occurrence of any major bleeding event. At 12 months, the primary end-point occurred in 9.8% of patients receiving ticagrelor as compared with 11.7% of those receiving clopidogrel (HR 0.84; 95% CI 0.77-0.92; *P*< 0.001). Similar figures were achieved by evaluating the rate of the primary outcome at 30 days (4.8% for ticagrelor group versus 5.4% for clopidogrel group; *P*= 0.045). The rate of death from any cause was also reduced with ticagrelor (4.5% versus 5.9% with clopidogrel; *P*< 0.001). Major, life-threatening, or fatal bleedings did not differ between those on ticagrelor and on clopidogrel (11.6% and 11.2%, respectively; *P*=0.43), nor did CABG-related major bleeding, although ticagrelor patients were allowed to undergo CABG earlier (24-72 hours), after withdrawing from the study drug, as compared to those on clopidogrel (5 days). However, Vis-à-vis fewer fatal bleeding episodes of other types, a higher rate of higher major bleeding not related to coronary artery bypass grafting (4.5% versus 3.8%; P=0.03), including fatal intracranial bleedings, were detected in subjects on ticagrelor. Secondary end-point evaluation showed significant differences in the rates of myocardial infarction (5.8% in the ticagrelor group versus 6.9% in the clopidogrel group; *P*= 0.005) and of death from vascular causes (4.0% versus 5.1%; *P*= 0.001), but not of stroke (1.5% versus 1.3%; *P*= 0.22).

More patients in the ticagrelor group discontinued the study drug due to adverse events compared to the clopidogrel group (7.4% versus 6.0%; *P*< 0.001). A significantly higher reversible increase in serum uric acid and creatinine was found in subjects receiving ticagrelor. Dyspnea, nausea, hypotension, and asymptomatic ventricular pauses were more frequent in the ticagrelor group, maybe because of an adenosine-mediated response (60). Thus, the use of ticagrelor should be evaluated with caution in patients with hyperuricemia, bradyar-rhythmias without pacemakers, and syncope and in those at high risk of bleeding (e.g. elderly, low body-weight, renal dysfunction) and avoided in patients with history of stroke (61). Of interest, in the ONSET/ OFFSET study, 123 patients with stable coronary artery disease receiving aspirin (75-100 mg/d) received on top ticagrelor (180-mg load, 90-mg b.i.d. maintenance dose (*n* = 57)), clopidogrel (600-mg load, 75-mg/d maintenance dose (*n* = 54)), or placebo (*n* = 12) for 6 weeks. Ticagrelor achieved a more rapid and greater platelet inhibition (evaluated with 20 μmol/L ADP) than did high-loading-dose clopidogrel. In addition, a faster offset occurred with ticagrelor than with clopidogrel (4-72-hour slope (% inhibition of platelet aggregation/h) -1.04 versus -0.48; *P*< 0.0001) (62). To evaluate further the safety and efficacy parameters, in a phase III study (NCT01294462) 90 mg/twice daily ticagrelor (on top of 100 mg aspirin) will be compared to 75 mg clopidogrel (on top of 100 mg aspirin as well) in ACS subjects with a planned PCI. The safety outcome will be evaluated by measuring the time to first occurrence of any major bleeding event. The time to first occurrence of the composite of death from any vascular cause (myocardial infarction and stroke) will also be measured.

The study is expected to enroll 800 subjects, and the estimated study completion date is August 2012. In addition, a randomized phase III PEGASUS trial (NCT01225562), on an expected number of 21,000 subjects with a history of myocardial infarction, will evaluate the efficacy of ticagrelor (90 or 60 mg/twice daily) compared to placebo on top of aspirin (100 mg) in the prevention of the composite end-point of cardiovascular death, non-fatal MI, or non-fatal stroke. The end of the study is expected by February 2014.

### Elinogrel

Elinogrel (PRT128) is a direct, reversible P_2_Y_12_ inhibitor available in both oral and intravenous formulations. In a randomized, double-blind, placebo-controlled trial (63) in which single intravenous doses (1–40 mg) were administered, elinogrel yielded a dose-dependent, complete inhibition of ADP-induced aggregation. All doses of elinogrel were well tolerated, with no serious adverse events observed.

### Sarpogrelate

Sarpogrelate is a selective inhibitor of 5-HT platelet receptors. In the S-ACCESS trial (64), 1,510 patients with recent cerebral infarction were randomly assigned to receive either sarpogrelate (100 mg t.i.d.) or aspirin (81 mg/day). Mean follow-up was 1.59 years, with the recurrence of a cerebral infarction as primary efficacy end-point. Clusters of serious vascular events (stroke, acute coronary syndrome, or vascular death) were secondary end-points. The aim of the primary efficacy analysis was the non-inferiority of sarpogrelate with respect to aspirin, the upper limit of the 95% CI of the hazard ratio for the recurrence of cerebral infarction not exceeding 1.33. Cerebral infarction recurred in 72 patients in the sarpogrelate group and in 58 in the aspirin group (HR 1.25; 95% CI 0.89-1.77; P=0.19). A serious vascular event occurred in 90 and in 85 patients, respectively (HR 1.07; 95% CI 0.80-1.44; *P*= 0.65). The overall incidence of bleeding events was 89 (11.9%) and 131 (17.3%), respectively

### DZ-697b

DZ-697b inhibits collagen and ristocetin-mediated platelet adhesion and aggregation and does not require metabolization to generate its active compound (65). The antithrombotic effect and the bleeding time prolongation of three DZ-697b doses were compared with 300 mg clopidogrel in 20 healthy subjects randomized to a single oral dose of DZ-697b (60, 120, and 360 mg) or clopidogrel (300 mg). DZ-697b (120 mg) showed antithrombotic effects comparable to 300 mg clopidogrel, with significantly reduced bleeding time prolongations.

### Atopaxar (E5555)

Atopaxar (E5555) is a small molecule, *1-(3-tert-butyl-4-methoxy-5-morpholinophenyl) -2- (5,6-diethoxy-7-fluoro-1 -intino-1,3-dihydro-2H-isoindol-2yl) ethanone hydrobromide* that acts as PAR-1 antagonist. In healthy volunteers, E5555 showed antiplatelet effects without increasing bleeding times. Two randomized, double-blind, placebo-controlled phase II clinical trials (J-LANCELOT) (66) have assessed the safety and tolerability of atopaxar in addition to standard therapy in patients with ACS or high-risk coronary artery disease. In addition to aspirin, patients received atopaxar (50 mg, 100 mg, or 200 mg) or placebo once daily for 12 or 24 weeks. All atopaxar doses tested achieved a significant level of platelet inhibition with no increase in severe bleeding episodes. The rate of major cardiovascular adverse events in the atopaxar group was similar to placebo (ACS 6.6% and 5.0% in placebo or E5555, respectively, P=0.73; CAD 4.5% placebo versus 1.0% E5555, P= 0.066).

### Vorapaxar

Vorapaxar (SCH 530348) is an orally active and reversible agent belonging to the class of thrombin receptor antagonists (TRAs). Vorapaxar blocks the platelet PAR-1 receptor, inhibiting thrombin-induced activation and aggregation of platelets without affecting thrombin-induced fibrin production. Phase I study data showed a significant dose-related inhibition of platelet aggregation with > 90% inhibition at 1 h, lasting at least 72 h in the absence of significant adverse events (67). The TRA-PCI (68), a randomized, double-blind, multicenter phase II study, was designed to assess tolerability and safety of an oral loading dose of vorapaxar (10 mg, 20 mg, or 40 mg) in patients undergoing coronary angiography with planned PCI. In addition to the standard of care (aspirin ± clopidogrel), those who subsequently underwent PCI received an oral maintenance dose (0.5 mg, 1.0 mg, or 2.5 mg/day) of vorapaxar or placebo for 60 days. The primary end-point was the incidence of clinically significant major or minor bleeding according to theTIMI scale. In spite of the limited time of observation (60 days) versus the very long half-life (100-360 h) of vorapaxar, no difference in the primary end-point of major or minor bleeding has been found (2%, 3%, 4% in the three loading dose groups versus 3.3% in placebo group;P= 0.57). Nor were significant differences in major cardiovascular events observed in the two groups. Platelet function testing showed a very potent and sustained effect with the 40-mg loading dose and the 2.5-mg maintenance dose. In a subgroup of subjects undergoing coronary bypass surgery while under vorapaxar, there was no evidence of increased bleeding, suggesting that PAR-1 blockade during cardiopulmonary bypass may ‘preserve’ normal hemostasis (69). In a phase II study (70), 117 NSTEMI patients undergoing non-urgent PCI have been randomized to receive, on top of standard dual antiplatelet therapy (aspirin + ticlopidine) and heparin, two different dosages of vorapaxar (20 mg or 40 mg loading dose, followed by 1 mg/d or 2.5 mg/d maintenance dose) or placebo. While TIMI major and minor bleedings were similar between the different treatment arms (14% versus 10%), peri-procedural AMI was significantly lower in treated patients (16.9% versus 42.9 in the placebo group %; *P*= 0.013).

Two large multicenter, international, prospective, double-blind, placebo-controlled phase III clinical trials were designed to establish the potential role of vorapaxar in treatment of CAD patients. TRA* CER (71) is a prospective, randomized, double-blind, multicenter phase III trial designed to determine whether vorapaxar (40-mg loading dose, followed by 2.5 mg daily for at least 1 year) can lead to further reduction in ischemic events when added to standard medical treatment in NSTEMI patients. As an estimate, 19,000 subjects are required to achieve adequate power. Primary composite end-point is the effect on cardiovascular death, AMI, stroke, recurrent ischemia with hospitalization, urgent PCI, and bleeding.

TRA 2°P-TIMI 50 (72) will evaluate the efficacy and safety of vorapaxar during long-term treatment of patients with established atherosclerotic disease receiving standard therapy (up to 27,000 patients). The study targeted patients with a documented history of AMI or stroke within 2 weeks to 12 months before the time of inclusion. Patients with peripheral artery disease (PAD) are eligible for inclusion if they have intermittent claudication in conjunction with an ankle-brachial index < 0.85 or previous revascularization for limb ischemia. Participants are randomized to receive vorapaxar (2.5 mg daily) or placebo in addition to standard therapy for at least 1 year. The primary composite end-point is cardiovascular death, AMI, stroke, or urgent coronary revascularization, whereas the evaluation of long-term safety includes bleeding events defined by the GUSTO and TIMI criteria.

## Future antiplatelet drugs

In addition to the antiplatelet drugs reported above, there is a continuous effort to identify newer targets toward which to direct pharmacological strategies.

Gas6 (growth arrest-specific gene 6), a member of the family of vitamin K-dependent proteins, is stored in platelet α granules and is released upon activation (73). Although it has a 44% sequence homology with protein S, it does not show any anticoagulant activity (74). Being a growth factor-like molecule, as it interacts with receptor tyrosine kinases (RTKs) of the Tyro3, Axl, and MerTK (TAM) family, Gas6 employs a unique mechanism of action, interacting through its vitamin K-dependent Gla module with phosphatidylserine-containing membranes and through its carboxy-terminal LG domains with the TAM membrane receptors. Studies indicate an association between Gas6 and stroke. Inhibition of Gas6 function can be achieved by Gas6-neutralizing antibodies, by proteases cleaving the extracellular domain of Gas6 receptors, or by inactivation of RNA for Gas6 or Gas6 receptors. Antibodies to the carboxy-terminal part of Gas6, responsible for the binding to its receptors, inhibit the aggregation of human platelets induced by ADP (75).

Matrix metalloproteinases (MMPs) are involved in tissue remodeling and in the progression of atherosclerosis (76). Human platelets contain MMP-2 in their cytosol and release them upon aggregation (77). Active MMP-2, potentiating phosphoinositide-3 kinase activation, amplifies platelet aggregation induced by a variety of agonists in an aspirin- or ADP-receptor antagonist-independent fashion (78). MMP-2-neutralizing antibodies have shown protective effects in hearts exposed to ischemia-reperfusion injury (79). MMP-2 gene-silencing techniques (80) may be exploited to gain cell-selective MMP-2 inhibition.

The CD40 ligand (CD40L; CD 154) is a trans-membrane protein expressed on the surface of activated platelets. Following its exposure, an exodomain (soluble CD40 ligand, sCD40L) is released and binds to integrin α_IIb_β_3_ on activated platelets, thus promoting thrombus stabilization (81). Therapeutic targeting of the CD40-CD40L axis using humanized anti-CD40L antibodies has already been attempted (82). Shedding of sCD40L from the surface of activated platelets can be prevented by an anti-CD40L antibody (G28-5). This may reduce plasma concentrations of sCD40L and, in turn, blunt platelet reactivity (83). In addition to the production by platelets, PGE_2_ is also produced by other cells in blood, including monocytes. Low concentrations of PGE_2_ potentiate platelet aggregation by priming protein kinase C activation and by inhibiting ade-nylylcyclase. The facilitating effects of PGE_2_ on platelets are mediated by EP3 (84,85). In animal studies, DG041, a novel first-in-class antagonist of EP3, effectively inhibits platelet aggregation without increasing the bleeding time (86). In a phase II trial in patients with peripheral arterial disease, DG041 was found to reduce platelet activation (87).

## Conclusions

Major limitations of current antiplatelet drugs include risk of bleeding, significant interindividual variability in the response, and extended duration of action that cannot be reversed if the need for hemostasis or emergency surgery arises. Newer anti-platelet drugs have addressed some but not all these limitations. Dual antiplatelet treatment has only been efficacious in acute coronary syndromes. In addition, newer thienopyridines did not show advantages over and above those of ticlopidine or clopidogrel as to reduction of stroke. Because of its pharmacodynamic characteristics, prasugrel is more efficacious in preventing ischemic events in patients with ACS undergoing PCI, but with increased bleeding complications. The same concept is true for ticagrelor. Platelet activation by thrombin is distinct and appears to be less important for hemostasis than fibrin generation (preclinical data). Accordingly, inhibition of PAR-1 function rather than inhibition of fibrin generation or activity may provide a newer strategy for treatment of thrombotic disorders in humans (88). Individual bleeding risk, however, has to be further defined, as this would be the first antiplatelet drug in which a greater efficacy is not associated with a worse safety (greater bleeding tendency).

A concerted effort that takes into consideration clinical, genetic, and laboratory information represents a major direction to be pursued in order to tailor the therapeutic approach for individual patients. The results of on-going clinical trials and the identification of further potentially useful targets for antiplatelet treatment (87) will hopefully help address this issue.

## Note

While this manuscript was under revision, results from LANCELOT-CAD were published (Circulation. 2011;123:1854-1863). In this study patients with a history of high-risk CAD were randomized to receive 50, 100, or 200 mg daily atopaxar or matching placebo for 24 weeks and followed up for an additional 4 weeks. Although no difference was found as to major bleeding events, overall bleeding incidence (key safety end point) showed a dose-dependent trend in the atopaxar arm as compared with placebo by CURE criteria (p for trend=0.01) but not by TIMI criteria (p for trend=0.07). As to the major adverse cardiac events occurrence (secondary end points), no significant difference was found between the atopaxar treatment arms and placebo (2.6% vs 4.6%; p=0.20). In addition, a transient elevation in liver transaminases and dose-dependent QTc prolongation without apparent complications were observed in higher-dose atopaxar treatment groups.
